# Alcohol-Related Risk, Depressive Symptoms, HRQoL, and Coping Phenotypes in Head-and-Neck Cancer: A Multicenter Cross-Sectional Study Stratified by Clinical Stage

**DOI:** 10.3390/medicina62040671

**Published:** 2026-04-01

**Authors:** Alexandru-Romulus Hut, Gheorghe Iovanescu, Eugen Radu Boia, Delia Ioana Horhat, Andrada Ioana Dumitru, Raphael Galant, Cosmin Rosca, Andreea Mihaela Kis, Nicolae Constantin Balica

**Affiliations:** 1 Doctoral School, “Victor Babes” University of Medicine and Pharmacy, Eftimie Murgu Square 2, 300041 Timisoara, Romania; alexandru.hut@umft.ro (A.-R.H.); andrada.dumitru@umft.ro (A.I.D.); balica@umft.ro (N.C.B.); 2 Department of Ear-Nose-Throat, Faculty of Medicine, “Victor Babes” University of Medicine and Pharmacy, Eftimie Murgu Square 2, 300041 Timisoara, Romania; giovanescu@umft.ro (G.I.); boia.eugen@umft.ro (E.R.B.); horhat.ioana@umft.ro (D.I.H.); 3 Lariboisiere University Hospital, University Paris Cité, Assistance Publique des Hôpitaux de Paris (AP-HP), 75015 Paris, France; raphaelumf@gmail.com; 4 U1123, National Institute of Health and Medical Research, University Paris Cité, 75006 Paris, France; 5 Oculens Clinic, Calea Turzii, No. 134-136, 400501 Cluj-Napoca, Romania; 6 Department of Management and Communication in Dental Medicine, Department I, Faculty of Dental Medicine, “Victor Babes” University of Medicine and Pharmacy, Eftimie Murgu Square 2, 300041 Timisoara, Romania

**Keywords:** head and neck neoplasms, alcohol drinking, alcoholism, depression, quality of life

## Abstract

*Background and Objectives*: Alcohol misuse, depressive symptoms, maladaptive coping, and impaired health-related quality of life (HRQoL) are clinically relevant in head-and-neck cancer, but their interrelationships are not fully captured by clinical stage alone. This multicenter study examined these domains at first admission and explored whether data-driven psychosocial phenotypes could complement stage-based stratification. *Materials and Methods*: In this multicenter cross-sectional study conducted at participating ENT/oncology centers in Timișoara and Oradea, Romania, during May 2024–October 2026, 64 adults with head-and-neck cancer completed the Alcohol Use Disorders Identification Test (AUDIT), Patient Health Questionnaire-9 (PHQ-9), EuroQol five-dimension questionnaire (EQ-5D), and Coping Orientation to Problems Experienced (COPE). Patients were compared by stage (I–III vs. IV). We also examined correlations, modeled poor HRQoL (EQ-5D health sum ≤ 9), derived COPE factor scores, identified psychosocial profiles by unsupervised clustering, and explored an indirect-association framework linking AUDIT, PHQ-9, and EQ-5D problem severity. *Results*: Stage IV disease was associated with greater tumor burden but not with worse psychosocial scores. Overall, 18.8% met criteria for possible alcohol dependence (AUDIT ≥ 20), and PHQ-9 scores correlated with poorer EQ-5D health status (ρ = −0.275; *p* = 0.028). Three psychosocial profiles showed significant differences in alcohol-risk burden, depressive symptoms, and coping signatures. The exploratory indirect-effect analysis did not support a statistically significant PHQ-9-mediated association between AUDIT and EQ-5D problem severity (indirect effect 0.002; 95% CI −0.018 to 0.022). *Conclusions*: Alcohol-related risk and depression-associated HRQoL burden were common and were not meaningfully explained by stage alone. Exploratory phenotype-based stratification may help identify patients who could benefit from earlier supportive-care triage.

## 1. Introduction

Head-and-neck cancer, including laryngeal cancer, remains a major survivorship challenge because both disease and treatment can permanently affect voice, swallowing, appearance, social participation, and day-to-day functioning [1,2]. According to the latest International Agency for Research on Cancer GLOBOCAN release, there were close to 20 million new cancer cases and 9.7 million cancer deaths worldwide in 2022 [1]. These figures support the need to complement anatomic staging with survivorship-focused assessment, particularly in tumor groups in which behavioral exposures and psychosocial burden can shape recovery trajectories [2].

Among the modifiable determinants of head-and-neck cancer outcomes, alcohol use is especially important because it is both a major etiologic exposure and a clinically actionable survivorship issue after diagnosis [3,4]. Dose–response evidence links alcohol consumption to cancers of the upper aerodigestive tract, while oncology societies increasingly recommend routine alcohol screening and counseling in cancer care pathways [3,4].

Importantly, continued alcohol exposure remains relevant after diagnosis. Studies in head-and-neck cancer survivorship show that a non-trivial subset of patients continue to drink at levels warranting intervention, and alcohol burden may coexist with poorer recovery trajectories even when tumor stage differs [5,6].

The Alcohol Use Disorders Identification Test (AUDIT) was developed as a brief, scalable instrument to identify hazardous and harmful drinking and to flag potential dependence using clinically interpretable cutoffs [7]. Importantly for busy oncology workflows, the AUDIT has demonstrated strong reliability when self-administered within broader health-risk questionnaires, supporting its use for rapid risk stratification and referral decisions [8].

Psychological distress, especially depressive symptoms, is also common in head-and-neck oncology and may be amplified by symptom burden, communication changes, stigma, uncertainty, and treatment intensity. Pragmatic tools such as the Patient Health Questionnaire-9 (PHQ-9) provide a validated severity measure that supports threshold-based triage and longitudinal monitoring in clinical and research settings [9].

Beyond alcohol use and depressive symptoms, coping responses may help explain why patients with similar disease extent and treatment histories report markedly different mental-health and HRQoL trajectories. COPE-based instruments capture heterogeneous behaviors such as active coping, planning, support seeking, denial, disengagement, and substance-related coping, and these domains can be summarized through data-reduction approaches that improve interpretability and reduce collinearity in multivariable models [10,11]. Clinically, such coping patterns may cluster with alcohol-risk burden and depressive symptoms, thereby supporting more integrated psychosocial triage rather than isolated single-domain referrals.

Patient-centered outcomes are increasingly prioritized in head-and-neck oncology, and HRQoL measurement should balance feasibility with clinical meaning. Disease-oriented instruments such as the EORTC QLQ-C30 have long provided standardized assessment of functional and symptom domains in oncology trials and observational studies [12]. Generic preference-based measures such as the EQ-5D enable concise profiling across mobility, self-care, usual activities, pain/discomfort, and anxiety/depression, supporting health-economic and comparative effectiveness perspectives [13]. The newer EQ-5D-5L improves descriptive sensitivity in many settings [14] and has shown validity in head-and-neck cancer populations when compared with disease-specific measures [15].

Accordingly, this study aimed to: (1) compare alcohol risk, depressive symptoms, EQ-5D–based HRQoL, and coping-factor scores between stage I–III and stage IV disease; (2) derive empirically interpretable coping dimensions from item-level COPE responses; (3) identify data-driven psychosocial profiles combining alcohol use, depression, coping, and HRQoL; and (4) explore whether depressive symptoms were statistically consistent with an indirect association between alcohol-risk severity and EQ-5D problem burden. We anticipated that psychosocial phenotypes would capture heterogeneity not explained by stage alone.

## 2. Materials and Methods

### 2.1. Study Design and Setting

We conducted a multicenter, observational, cross-sectional study within the Doctoral School of the “Victor Babeș” University of Medicine and Pharmacy Timișoara, in collaboration with affiliated ENT/oncology services in Timișoara and Oradea, Romania. Recruitment and questionnaire administration were performed during May 2024–October 2026 in routine outpatient and planned inpatient pathways using a standardized protocol across participating centers.

The primary endpoint set comprised AUDIT, PHQ-9, EQ-5D, and COPE-derived scores analyzed in relation to clinical stage. Secondary analyses explored coping-factor structure, psychosocial clustering, and an exploratory indirect-effect model linking alcohol risk, depressive symptoms, and EQ-5D problem burden.

### 2.2. Participants: Eligibility, Recruitment, and Clinical Characterization

Adults (≥18 years) with a confirmed diagnosis of head-and-neck cancer and documented clinical staging at the time of psychosocial assessment were eligible. Patients were required to be able to complete questionnaires independently or with minimal assistance (e.g., reading support without interpretive guidance). We excluded patients who: (i) were unable to provide informed consent; (ii) had severe cognitive impairment, acute psychosis, or other conditions preventing valid self-report; (iii) were receiving emergent resuscitative care at the time of approach; or (iv) had substantial missing questionnaire data precluding scoring of the primary instruments.

Consecutive sampling was used in participating clinics/wards during the study period. A trained study team member screened clinic schedules and ward lists for potentially eligible patients, confirmed eligibility via chart review (diagnosis and stage), and invited patients to participate. To reduce social desirability bias and preserve confidentiality, patients completed self-report measures in a private setting; sealed paper forms or secure electronic entries were used depending on site logistics.

Tumor characteristics were extracted from medical records by trained abstractors using a harmonized data dictionary. Variables included: age, sex, residence (urban/rural), primary tumor category (T), nodal status (N), and distant metastasis (M). Stage grouping was defined a priori as stage I–III versus stage IV based on the treating team’s documented staging in the clinical record (with TNM elements retained as separate variables for descriptive reporting and sensitivity checks). When multiple staging notes existed, the closest staging assessment to the questionnaire date was used.

Consecutive eligible patients were invited during the recruitment period. The analytic cohort comprised 64 participants with sufficient data to score the primary patient-reported outcome measures. Participants with incomplete responses preventing valid score calculation were excluded from score-specific analyses and from complete-case multivariable models, as appropriate. Because cross-site screening logs were not harmonized prospectively, a formal overall response rate could not be calculated retrospectively with certainty; this is now acknowledged as a limitation.

No formal a priori sample-size calculation was performed because this study was conceived as an exploratory cross-sectional analysis of consecutively presenting patients. Accordingly, regression, clustering, and indirect-effect analyses should be interpreted as hypothesis-generating.

### 2.3. Measures and Study Variables

All patient-reported outcome measures (PROMs) were administered in Romanian, in person, using standardized self-completion forms distributed by trained study personnel at the index assessment. When necessary, neutral reading assistance was permitted without interpretive coaching. Scores were calculated according to the original instrument structure and published scoring rules, with directionality prespecified before analysis [7,8,9,10,11,12,13,14,15].

Alcohol use was measured with the Alcohol Use Disorders Identification Test (AUDIT), a validated screening tool for hazardous, harmful, and dependent drinking [7,8]. AUDIT totals were analyzed continuously and categorized using conventional cut points: low risk (0–7), hazardous use (8–15), harmful use (16–19), and possible dependence (≥20). For selected analyses, we also used a binary indicator of possible dependence (AUDIT ≥ 20).

Depressive symptom severity was assessed with the Patient Health Questionnaire-9 (PHQ-9), a validated brief measure of depressive symptom burden [9]. PHQ-9 totals were analyzed continuously and categorized as minimal–mild (<10), moderate (10–14), and moderate-severe–severe (≥15). A threshold of PHQ-9 ≥ 10 was used in subgroup analyses to indicate at least moderate depressive symptom burden.

Health-related quality of life was evaluated with the EuroQol EQ-5D descriptive system [13,14,15]. To make score direction explicit throughout the manuscript, we used two complementary summaries: an EQ-5D health sum, where higher values indicate better overall health in the present analytic convention, and an EQ-5D problem-severity sum, where higher values indicate worse HRQoL burden.

We additionally recorded the EQ-5D anxiety/depression dimension level and derived a limitation count (number of dimensions with scores < 3) to summarize multidomain impairment in a clinically interpretable way. “Poor HRQoL” was defined a priori as an EQ-5D health sum ≤ 9 for logistic-regression modeling.

Coping was assessed at item level with the COPE framework [10,11]. Because several coping subdomains are conceptually overlapping, we used exploratory dimension reduction to derive empirically supported factor scores for subsequent analyses. For clinical interpretation of the retained solution, Factor 1 predominantly reflected active/adaptive coping content (e.g., active coping, planning, positive reframing, and support seeking), whereas Factor 2 predominantly reflected avoidant/substance-linked content (e.g., denial, behavioral disengagement, and substance-related coping).

Prespecified covariates included age, sex, disease stage group (I–III vs. IV), residence (urban vs. rural), and derived coping factor scores. These were selected based on clinical plausibility and the need to control for potential confounding in multivariable and mediation models while maintaining parsimony given cohort size.

### 2.4. Data Collection Procedures, Quality Control, and Missing Data

PROMs were administered at a single timepoint per participant, targeted to coincide with a scheduled clinic visit or inpatient evaluation to reflect the patient’s contemporary psychosocial status. Clinical variables (TNM/stage) were abstracted for the same index time window to ensure alignment between disease status and PROM responses.

All participating centers used the same case report form structure and data dictionary. Study personnel were trained on: (i) neutral questionnaire administration; (ii) handling patient questions without coaching responses; (iii) completeness checks; and (iv) chart abstraction rules for tumor variables. Periodic cross-site checks were performed on a subset of records to confirm consistent coding of key variables (stage group, T/N/M categories, and urban/rural residence).

Paper forms (if used) were double-entered by two independent operators; discrepancies were resolved by referring to the original document. Electronic data (if used) were entered into a password-protected database with restricted access. Participants were assigned unique study identifiers; the linkage file was stored separately from analytic datasets.

We summarized missingness by instrument and by item. For COPE item-level analyses, items were screened for missingness; when missingness was low and judged compatible with an ignorable mechanism, we used item-wise median imputation prior to standardization and dimension reduction. For AUDIT, PHQ-9, and EQ-5D, scoring followed instrument rules; participants with insufficient items to compute valid totals were excluded from analyses requiring that score (pairwise deletion for correlation matrices; complete-case for multivariable models unless otherwise stated). The missing-data strategy was predefined to preserve interpretability while limiting bias from excessive imputation in a modest sample.

### 2.5. Statistical Analysis

Statistical analyses were performed in SPSS v27.0. Because this study was exploratory and the analytic sample was modest, all multivariable, clustering, and indirect-effect analyses were prespecified as hypothesis-generating rather than confirmatory.

Continuous variables were summarized as mean ± standard deviation or median [interquartile range], according to distribution, and categorical variables as *n* (%). Before parametric testing, assumptions were checked using visual distribution review together with formal assessment of normality and variance homogeneity. Student’s *t* test or analysis of variance was used only when assumptions were acceptable; otherwise, Mann–Whitney U, Kruskal–Wallis, or Welch-corrected procedures were applied. Categorical comparisons used χ^2^ or Fisher’s exact tests, as appropriate.

Bivariate associations among psychosocial measures and age were assessed using Spearman rank correlations (ρ). Predictors of poor HRQoL (EQ-5D health sum ≤ 9) were evaluated with multivariable logistic regression using prespecified covariates. For regression diagnostics, we reviewed correlation structure and multicollinearity indices before final model specification.

COPE item structure was explored using principal component analysis with rotation after confirming factorability. Standardized factor scores were then carried forward into correlation, clustering, regression, and indirect-effect analyses. Internal consistency was summarized for the retained dimensions.

Psychosocial phenotypes (“cluster profiles”) were prespecified as a secondary objective. Clustering used standardized patient-level AUDIT, PHQ-9, EQ-5D problem-severity, and coping-factor scores, and the retained three-cluster solution was selected on the basis of internal fit, minimum cluster size, and clinical interpretability. The indirect-effect analysis was conducted with bootstrap resampling (5000 draws) and is reported using associative rather than causal language.

## 3. Results

### 3.1. Sample Characteristics and Stage Distribution

This study included 64 patients with head-and-neck cancer (mean age 65.30 ± 8.44 years), predominantly male (58/64, 90.6%). Most had stage IV disease (48/64, 75.0%), with a cohort profile enriched for locoregionally advanced tumor burden. This descriptive context is important because subsequent psychosocial comparisons were not stage-differentiated despite marked anatomic differences.

Table 1 summarizes baseline demographic and tumor features by stage group. Age, sex distribution, and residence were similar between groups, whereas T4 primaries and nodal involvement were, as expected, concentrated in stage IV disease.

### 3.2. Stage-Based Psychosocial Comparisons

Table 2 shows that alcohol-risk burden, depressive symptoms, EQ-5D health status, and coping-factor scores were similar in stage I–III and stage IV disease. In other words, it stage-differentiated tumor burden, but it did not meaningfully differentiate the psychosocial measures examined here.

### 3.3. Alcohol-Risk and Depressive-Symptom Strata

Table 3 details alcohol-risk strata across the full cohort and by stage and sex. Overall, 18.8% met AUDIT criteria for possible dependence, with no stage association. The apparent sex difference should be interpreted cautiously because only six women were included in the cohort.

Table 4 stratifies participants by PHQ-9 severity. Although between-group differences were not statistically significant, worsening PHQ-9 category corresponded to numerically lower EQ-5D health-sum values and a higher proportion of poor HRQoL, supporting the clinical relevance of depressive symptom burden even in a modest sample.

### 3.4. Correlation Structure and Multivariable HRQoL Model

Table 5 presents the bivariate correlation structure. The clearest signals were a modest positive association between AUDIT and COPE Factor 2 and an inverse association between PHQ-9 and EQ-5D health sum, consistent with poorer HRQoL at higher depressive-symptom burden.

Table 6 reports the multivariable model for poor HRQoL (EQ-5D health sum ≤ 9). No predictor reached conventional statistical significance, although stage IV disease showed a borderline inverse association with poor HRQoL that is considered exploratory and is discussed further below.

### 3.5. Cluster-Derived Psychosocial Phenotypes

Table 7 describes three cluster-derived psychosocial profiles based on AUDIT, PHQ-9, EQ-5D problem burden, and coping factors. The profiles were separated primarily by alcohol-risk burden, depressive-symptom severity, and coping signatures, whereas EQ-5D problem severity was less discriminative.

Taken together, the retained clusters suggest clinically recognizable constellations of lower-depression/more adaptive coping, high alcohol-risk with mixed psychological burden, and very high alcohol-risk with avoidant/substance-linked coping. These profiles should be viewed as exploratory and hypothesis-generating.

### 3.6. Exploratory Indirect-Effect Analysis and Supplementary Visual Summaries

Table 8 reports the exploratory indirect-effect analysis assessing whether the association between AUDIT and EQ-5D problem severity was statistically consistent with an indirect pathway through PHQ-9. The path, direct effect, total effect, and bootstrap-estimated indirect effect were all small and non-significant, with the indirect-effect confidence interval crossing zero.

**Figure 1:**
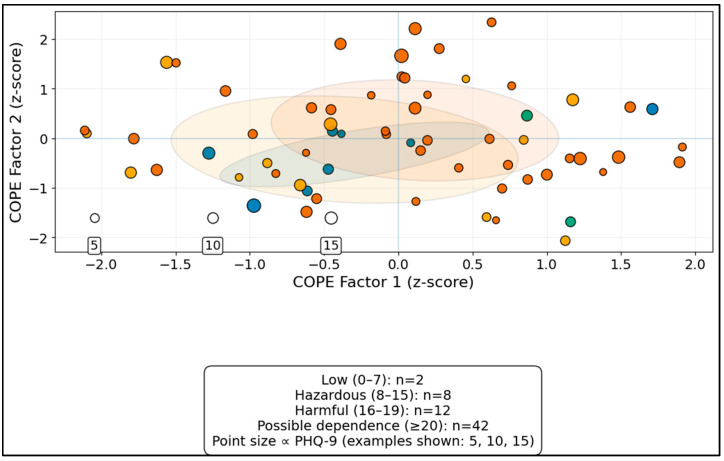
COPE factor space by AUDIT category.

Figure 1 complements the categorical AUDIT results by mapping patients in the coping-factor space. In the 64 complete cases, possible dependence (AUDIT ≥ 20) was present in 12/64 (18.8%); the AUDIT ≥ 20 group showed a non-significant trend toward higher COPE Factor 2 values, while PHQ-9 burden did not differ significantly, consistent with the weak AUDIT–PHQ-9 association observed in Table 5.

**Figure 2:**
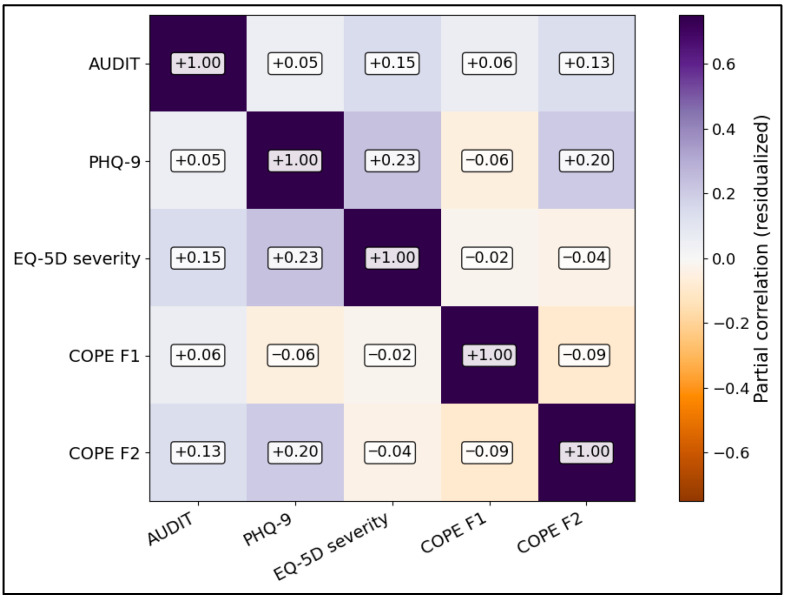
Partial-correlation heatmap adjusted for age, sex, and stage IV.

Figure 2 shows the covariate-adjusted partial-correlation structure after residualizing for age, sex, and advanced stage. The remaining associations were modest and exploratory, with the largest adjusted signal again observed between PHQ-9 and EQ-5D severity.

**Figure 3:**
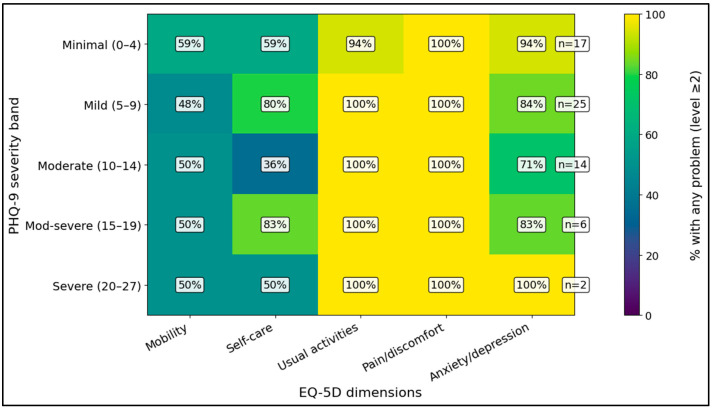
EQ-5D problem prevalence by PHQ-9 severity band.

Figure 3 summarizes EQ-5D problem prevalence across PHQ-9 strata. Pain/discomfort and usual-activity limitations were highly prevalent in all depression bands, whereas mobility and self-care showed greater variability, suggesting that the dominant generic HRQoL burden in this cohort centered on pain and daily functioning rather than on uniform impairment across all domains.

## 4. Discussion

### 4.1. Analysis of Findings

Despite the expected biologic gradient in tumor burden, we observed no meaningful stage-group differences in alcohol risk, depressive symptoms, generic HRQoL, or coping-factor scores at first admission. This pattern is consistent with the broader literature showing that patient-reported outcomes in head-and-neck cancer do not map linearly onto stage alone and may remain highly influenced by symptom perception, social context, comorbidity, and selection effects [16,17,18,19,20,21,22,23,24,25]. Recent European data likewise suggest that stage captures only part of the patient-reported burden, with several quality-of-life domains varying independently of anatomic extent [25].

A key clinical signal in our cohort is the high prevalence of clinically actionable alcohol risk: 18.8% met criteria for possible dependence, and a further 35.9% fell into hazardous or harmful categories. This finding reinforces the idea that alcohol burden in head-and-neck cancer behaves as a survivorship liability rather than simply a correlate of advanced stage. Patients with head-and-neck cancer are uniquely vulnerable because the same exposure that contributes to carcinogenesis may continue after diagnosis, may cluster with tobacco use and maladaptive coping, and may complicate symptom control, rehabilitation, and follow-up engagement [18,19,26].

Although PHQ-9 subgroup comparisons did not reach conventional significance for all EQ-5D outcomes, depressive symptom severity correlated with worse HRQoL, and the direction of the multivariable model remained clinically coherent. This interpretation is also in keeping with contemporary evidence that depression is common and may be under-recognized in head-and-neck cancer populations [21,22].

Our coping results add nuance. The modest positive association between AUDIT and COPE Factor 2 suggests that heavier alcohol-risk burden may cluster with a more avoidant or substance-linked coping signature, whereas the phenotype analysis showed that more adaptive coping was concentrated in the lower-depression cluster. These observations are directionally concordant with prior head-and-neck oncology work linking coping style to distress and quality of life [23,24].

Finally, the cluster-derived psychosocial phenotypes provide a clinically interpretable bridge between measurement and action. The borderline inverse association between stage IV disease and poor HRQoL in the multivariable model, although not statistically significant, may reflect selection and adaptation effects: patients able to attend first-admission assessment despite advanced disease may already represent a psychologically coping subgroup, and survivor bias cannot be excluded. At the same time, the null indirect-effect analysis should not be over-interpreted as evidence of absence, because the modest sample may have limited power to detect small indirect associations.

These findings support embedding routine, stage-independent psychosocial screening at head-and-neck cancer intake: nearly 1 in 5 patients met criteria for probable alcohol dependence, and depressive symptoms tracked with worse HRQoL. Because the stage did not differentiate AUDIT/PHQ-9/EQ-5D distributions, relying on oncologic staging alone may miss high-need patients. A pragmatic pathway is: brief alcohol screening (AUDIT or AUDIT-C followed by full AUDIT when positive) plus depression screening (PHQ-2/PHQ-9) with tiered referral (behavioral oncology, addiction medicine, social work), and targeted coping-focused interventions aligned to the cluster-derived profiles (high alcohol-risk with low adaptive coping vs. low depression/adaptive coping). Nevertheless, these findings should be interpreted in light of potential residual confounding from unmeasured or incompletely controlled factors, including underlying comorbidities and other patient- and treatment-related characteristics [27,28,29,30,31].

### 4.2. Study Limitations

This study also has several strengths, including its multicenter design, simultaneous assessment of alcohol use, depressive symptoms, generic HRQoL, and coping, and its integration of conventional comparative analyses with data-driven exploratory methods. Nevertheless, several limitations warrant emphasis. The cross-sectional design precludes temporal or causal inference. The sample was modest, with very few women, which limits sex-stratified conclusions and means the observed male predominance in AUDIT ≥ 20 should not be over-generalized. No formal a priori sample-size calculation was performed, so the regression, clustering, and indirect-effect analyses should be interpreted as exploratory and potentially underpowered, with possible Type II error. All measures were self-reported and therefore susceptible to social desirability and recall bias. EQ-5D is a generic rather than head-and-neck-specific HRQoL instrument, so it may underrepresent domains such as speech and swallowing. Finally, the COPE factor solution and cluster profiles require external validation in larger longitudinal cohorts.

### 4.3. Future Research Directions

Future work should prospectively validate these psychosocial profiles in larger, sex-balanced, multicenter cohorts with repeated assessments across diagnosis, treatment, and survivorship. Studies that combine generic HRQoL instruments with head-and-neck-specific measures and objective treatment/toxicity markers may clarify whether early alcohol-risk and coping phenotypes can improve referral pathways, adherence, and longer-term functional outcomes.

## 5. Conclusions

In this multicenter head-and-neck cancer cohort, alcohol-related risk and depressive symptoms were prevalent and clinically meaningful, yet they did not differ by clinical stage despite clear stage-related differences in tumor burden. Probable alcohol dependence affected nearly one fifth of the cohort, depressive symptoms tracked with worse HRQoL, and exploratory phenotype analysis identified heterogeneity not captured by stage alone. These findings support routine stage-independent psychosocial screening at intake and suggest that phenotype-informed supportive-care triage deserves prospective validation.

## Figures and Tables

**Table 1:** Demographic and tumor characteristics by stage group (I–III vs. IV).

Variable	Stage I–III (*n* = 16)	Stage IV (*n* = 48)	*p*-Value
Age, years	65.38 ± 7.91	65.27 ± 8.65	0.963
Male sex	14 (87.5%)	44 (91.7%)	0.625
Urban residence	7 (43.8%)	23 (47.9%)	0.77
T4 primary tumor	5 (31.2%)	45 (93.8%)	<0.001
N+ (nodal involvement)	0 (0.0%)	40 (83.3%)	<0.001
Distant metastasis (M1)	0 (0.0%)	1 (2.1%)	>0.999

I–III, stages I to III; IV, stage IV; T4, tumor stage 4 primary; N+, nodal involvement present; M1, distant metastasis present.

**Table 2:** Questionnaire outcomes and EQ-5D limitations by stage group.

Measure	Stage I–III (*n* = 16)	Stage IV (*n* = 48)	*p*-Value
AUDIT score	7.0 [6.0–10.0]	8.0 [5.0–12.0]	0.698
PHQ-9 score	6.5 [3.8–11.0]	8.0 [4.0–10.2]	0.945
EQ-5D health sum (higher = better)	10.19 ± 1.56	10.46 ± 1.75	0.555
EQ-5D limitation count (digits < 3)	2.0 [2.0–3.0]	2.0 [2.0–3.0]	0.906
COPE factor 1 (PCA score)	0.25 ± 0.93	−0.08 ± 1.02	0.25
COPE factor 2 (PCA score)	−0.05 ± 0.89	0.02 ± 1.04	0.571

AUDIT, Alcohol Use Disorders Identification Test; PHQ-9, Patient Health Questionnaire-9; EQ-5D, EuroQol 5-Dimension questionnaire; PCA, principal component analysis.

**Table 3:** AUDIT risk categories by stage and sex.

AUDIT Risk Category	Total (N = 64)	Stage I–III (*n* = 16)	Stage IV (*n* = 48)	Male (*n* = 58)	Female (*n* = 6)
Low risk (0–7)	29 (45.3%)	7 (43.8%)	22 (45.8%)	24 (41.4%)	5 (83.3%)
Hazardous (8–15)	18 (28.1%)	5 (31.2%)	13 (27.1%)	16 (27.6%)	2 (33.3%)
Harmful (16–19)	5 (7.8%)	1 (6.2%)	4 (8.3%)	5 (8.6%)	0 (0.0%)
Possible dependence (≥20)	12 (18.8%)	3 (18.8%)	9 (18.8%)	12 (20.7%)	0 (0.0%)

Association of AUDIT categories with stage (χ^2^ *p* = 0.989); possible dependence (AUDIT ≥ 20) vs. stage (Fisher *p* = 0.715); possible dependence vs. sex (Fisher *p* = 0.006); AUDIT, Alcohol Use Disorders Identification Test; I–III, stages I to III; IV, stage IV.

**Table 4:** Depression severity subgroups (PHQ-9) and relationships with HRQoL, alcohol risk, and coping.

PHQ-9 Group	*n*	EQ-5D Health Sum (Higher = Better)	EQ-5D Anxiety/Depression Level (Median [IQR])	AUDIT (Median [IQR])	COPE_F2 (Mean ± SD)	Poor HRQoL (EQ-5D Sum ≤ 9), *n* (%)
Minimal–mild (<10)	43	10.70 ± 1.63	2.0 [2.0–3.0]	8.0 [5.0–12.0]	0.00 ± 1.05	6 (14.0%)
Moderate (10–14)	12	9.83 ± 1.70	2.0 [1.0–2.0]	7.5 [6.0–10.8]	0.02 ± 0.93	5 (41.7%)
Mod-severe–severe (≥15)	9	9.78 ± 1.48	2.0 [2.0–2.0]	9.0 [5.0–12.0]	−0.13 ± 1.16	3 (33.3%)

EQ-5D health sum across PHQ-9 groups (Kruskal–Wallis *p* = 0.116); AUDIT across PHQ-9 groups (*p* = 0.721); COPE Factor 2 across PHQ-9 groups (*p* = 0.866); poor HRQoL prevalence across PHQ-9 groups (χ^2^ *p* = 0.297). EQ-5D health-sum values are presented so that higher values indicate better health status. PHQ-9, Patient Health Questionnaire-9; EQ-5D, EuroQol five-dimension questionnaire; HRQoL, health-related quality of life; AUDIT, Alcohol Use Disorders Identification Test; IQR, interquartile range; and SD, standard deviation.

**Table 5:** Spearman correlations among AUDIT, PHQ-9, EQ-5D, coping factors, and age.

Variable 1	Variable 2	Spearman ρ	*p*-Value
AUDIT	PHQ9	0.129	0.311
AUDIT	EQ-5D_health_sum	0.212	0.092
AUDIT	COPE_F1	−0.156	0.218
AUDIT	COPE_F2	0.263	0.036
AUDIT	Age	−0.009	0.942
PHQ9	EQ-5D_health_sum	−0.275	0.028
PHQ9	COPE_F1	0.001	0.996
PHQ9	COPE_F2	−0.182	0.151
PHQ9	Age	0.001	0.992
EQ-5D_health_sum	COPE_F1	−0.101	0.427
EQ-5D_health_sum	COPE_F2	0.061	0.632
EQ-5D_health_sum	Age	0.231	0.066
COPE_F1	COPE_F2	0	0.999
COPE_F1	Age	−0.114	0.369
COPE_F2	Age	0.097	0.444

AUDIT, Alcohol Use Disorders Identification Test; PHQ-9, Patient Health Questionnaire-9; EQ-5D, EuroQol 5-Dimension questionnaire; and ρ, Spearman rank correlation coefficient.

**Table 6:** Multivariable logistic regression predicting poor HRQoL (EQ-5D health sum ≤ 9).

Predictor	aOR (95% CI)	*p*-Value
PHQ-9	1.12 (0.96–1.30)	0.137
AUDIT	0.97 (0.89–1.06)	0.52
StageIV	0.20 (0.04–1.10)	0.065
Age	0.98 (0.92–1.05)	0.572
Urban	0.90 (0.29–2.85)	0.861
COPE_F2_z	0.61 (0.27–1.40)	0.255

Stage IV compares stage IV vs. I–III; Urban compares urban vs. rural; COPE_F2_z is per + 1 SD increase in coping factor 2; HRQoL, health-related quality of life; EQ-5D, EuroQol five-dimension questionnaire; in this manuscript, higher EQ-5D health-sum values indicate better health status; aOR, adjusted odds ratio; CI, confidence interval; PHQ-9, Patient Health Questionnaire-9; AUDIT, Alcohol Use Disorders Identification Test.

**Table 7:** Data-driven psychosocial phenotypes (cluster profiles) using AUDIT + PHQ-9 + EQ-5D + COPE factors.

Variable	Profile 1	Profile 2	Profile 3	*p*-Value
AUDIT score	20.32 ± 6.14	16.62 ± 7.17	27.77 ± 4.77	<0.001
PHQ-9 total	9.18 ± 5.01	4.69 ± 3.36	9.08 ± 4.59	0.003
EQ-5D problem-severity sum (0 = best)	4.59 ± 1.26	4.06 ± 1.69	5.00 ± 1.10	0.186
COPE Factor 1 (z)	−0.55 ± 0.70	0.81 ± 0.70	−0.25 ± 0.65	<0.001
COPE Factor 2 (z)	−0.03 ± 0.89	−0.58 ± 0.44	0.65 ± 0.55	<0.001
AUDIT ≥ 20 (probable dependence)	18/22 (81.8%)	9/26 (34.6%)	16/16 (100.0%)	<0.001
PHQ-9 ≥ 10 (moderate+ depression)	10/22 (45.5%)	2/26 (7.7%)	7/16 (43.8%)	0.005
EQ-5D severity ≥ 6 (serious problems)	6/22 (27.3%)	5/26 (19.2%)	6/16 (37.5%)	0.418
Advanced stage (STD 3–4)	22/22 (100.0%)	21/26 (80.8%)	13/16 (81.2%)	0.038
Metastatic disease (M1)	0/22 (0.0%)	2/26 (7.7%)	0/16 (0.0%)	0.267

AUDIT, Alcohol Use Disorders Identification Test; PHQ-9, Patient Health Questionnaire-9; EQ-5D, EuroQol 5-Dimension questionnaire; COPE, Coping Orientation to Problems Experienced; z, standardized z-score; and M1, distant metastasis present.

**Table 8:** Covariate-adjusted mediation: AUDIT → depression (PHQ-9) → EQ-5D problem severity.

Path	Estimate (β)	SE	*p*	95% CI
a: AUDIT → PHQ-9	0.027	0.103	0.791	—
b: PHQ-9 → EQ-5D severity	0.072	0.037	0.055	—
c′: AUDIT → EQ-5D severity (direct)	0.03	0.028	0.29	—
c: AUDIT → EQ-5D severity (total)	0.032	0.029	0.272	—
Indirect effect (a × b), bootstrap	0.002	—	—	[−0.018, 0.022]

Outcome = EQ-5D problem-severity sum (0 = best; higher = worse). Bootstrap B = 5000 for the indirect effect; AUDIT, Alcohol Use Disorders Identification Test; PHQ-9, Patient Health Questionnaire-9; EQ-5D, EuroQol 5-Dimension questionnaire; β, regression coefficient; SE, standard error; CI, confidence interval; c′, direct effect; c, total effect; a × b, indirect (mediated) effect.

## Data Availability

The data presented in this study are available upon request from the corresponding authors.

## Abbreviations

AUDIT, Alcohol Use Disorders Identification Test; CI, confidence interval; COPE, Coping Orientation to Problems Experienced; EQ-5D, EuroQol five-dimension questionnaire; HRQoL, health-related quality of life; IQR, interquartile range; PCA, principal component analysis; PHQ-9, Patient Health Questionnaire-9; PROMs, patient-reported outcome measures; and SD, standard deviation.
